# Baby Boomers Who Provide Informal Care for People Living with Dementia in the Community

**DOI:** 10.3390/ijerph18189694

**Published:** 2021-09-15

**Authors:** Christina E. Miyawaki, Erin D. Bouldin, Christopher A. Taylor, Lisa C. McGuire

**Affiliations:** 1 Graduate College of Social Work, University of Houston, 3511 Cullen Boulevard, Room 110HA, Houston, TX 77204-4013, USA; 2 Department of Health & Exercise Science, Appalachian State University, 1179 State Farm Rd, Suite 432, P.O. Box 32071, Boone, NC 28608, USA; bouldinel@appstate.edu; 3 Alzheimer’s Disease & Healthy Aging Program, Centers for Disease Control and Prevention, 4770 Buford Hwy NE, Atlanta, GA 30341, USA; iyq3@cdc.gov (C.A.T.); cqu6@cdc.gov (L.C.M.)

**Keywords:** Alzheimer’s, baby boomers, Behavioral Risk Factor Surveillance System (BRFSS), caregivers, dementia, persons with dementia (PWD)

## Abstract

One in four Baby Boomers fills the informal caregiver role in the United States. The objectives of this study were to estimate the prevalence of Baby Boomers who are informal caregivers for people living with dementia and compare their physical and mental health status to caregivers for persons with conditions other than dementia using 2015–2018 Behavioral Risk Factor Surveillance System data (*N* = 10,602). We identified caregiving status (assisting a family member/friend with a long-term illness or disability in the past month, managing personal care, and not caring for a child/grandchild) and whether the care recipient’s major health condition was dementia. We calculated weighted estimates and used chi-square tests and log-binomial regression for comparisons of selected characteristics. Among Baby Boomer caregivers, 15.4% were caring for someone with dementia. Dementia caregivers were more likely to be female, caring for a parent/parent-in-law, and providing care longer than caregivers for persons without dementia. After adjusting for sociodemographic and caregiving characteristics, the prevalence of fair/poor health, frequent mental distress, and chronic conditions were similar across types of caregivers. Although no differences in caregiver’s physical and mental health by care recipient’s dementia status were found, we should underscore the importance of maintaining Baby Boomer caregivers’ health and well-being.

## 1. Introduction

More than 16 million people in the United States live with cognitive impairment [1] and approximately 6.2 million people aged 65 and older are reported to live with Alzheimer’s dementia [2]. The prevalence of Alzheimer’s disease increases with age from 5.3% (aged 65–74 years), to 13.8% (75–84 years), and 34.6% (aged 85 years and older) [2].

Informal caregivers—family members and friends who attend to another person’s health needs and provide unpaid assistance in the community [3]—frequently provide help to people living with a cognitive impairment or dementia [3,4,5]. Most caregivers of persons with dementia (PWD) provide support to a parent or spouse [3]. The majority of PWD (66%) live in the community and are cared for by informal caregivers, because PWD, like other aging adults, typically want to remain at home [6]. Informal caregivers can assist with this preference by providing in-home assistance and augment the healthcare system for the benefit of their care recipients. In addition, this caregiving supplements the health care system. If informal caregivers were not providing care to care recipients at home, the health care system would have to provide the care, and by providing this unpaid caregiving, it saves a substantial amount of health care cost [7].

The average age of informal caregivers is 49.2 years old [8], but one-third of caregivers are 65 and older [9]. Baby Boomers were born between 1946 and 1964 [10]. One in four Baby Boomers fills the informal caregiver role [7]. Compared with previous generations, Baby Boomers tend to have higher education levels with longer working years, lower marriage rates with fewer children or delayed childbirth, but higher separation or divorce rates [11,12,13]. Given the longer life expectancy of the Baby Boomer generation’s aging parents, this generation may be providing care and financial assistance longer than previous generations (nearly 40 years) and for multiple generations simultaneously with their children or young adult children [14]. Despite these characteristics, few studies have been conducted about Baby Boomer caregivers, particularly those caring for PWD [7,14]. Thus, it is important to understand more about Baby Boomers who are caregivers of PWD and describe their caregiving situations. The objectives of the study were to estimate the prevalence of Baby Boomer caregivers of PWD, describe their characteristics, and compare their physical and mental health to caregivers of persons with other conditions (PWOC). We hypothesized that Baby Boomers who were caregivers of PWD would have poorer physical and mental health compared to caregivers of PWOC.

## 2. Methods

### 2.1. Data Source

We used data from 46 jurisdictions included in the 2015–2018 Behavioral Risk Factor Surveillance System (BRFSS). The BRFSS is a telephone survey of non-institutionalized adults aged 18 years and older conducted annually by U.S. jurisdictions in partnership with the Centers for Disease Control and Prevention [15]. The BRFSS includes a core set of questions about health and demographic characteristics, and each jurisdiction may choose to include optional modules, one of which is the Caregiver Module. Between 2015 and 2018, 44 states, the District of Columbia, and Puerto Rico administered the Caregiver Module at least once. When jurisdictions administered the module in more than one year, we used only the most recent year of data in the analytic dataset; therefore, no jurisdiction is represented more than once in the analysis. This study was reviewed by the author’s university and classified as exempt (#19-0180). Overall median response rates for BRFSS were 47.2% in 2015, 47.0% in 2016, 45.1% in 2017, and 49.9% in 2018, which are similar to or better than other similar health surveillance systems (see Summary Data Quality Reports for each year at https://www.cdc.gov/brfss/annual_data/annual_data.htm, accessed on 11 September 2021). For the Caregiver Module, the total number of adult respondents aged ≥18 years was 108,995 in 2015, 76,593 in 2016, 24,388 in 2017, and 15,201 in 2018. The variability in sample size across years is a result of the number of jurisdictions choosing to include the module on their survey each year and different sample sizes within states.

### 2.2. Variables

Baby Boomers were defined as people born between 1946 and 1964 [10]. Because date of birth is not included in the BRFSS, we used self-reported age to categorize respondents as Baby Boomers: 50–69 years old in 2015, 51–70 years old in 2016, 52–71 years old in 2017, and 53–72 years old in 2018. We classified respondents as caregivers if they reported providing regular care or assistance during the past 30 days to a friend or family member who had a health problem or disability, caring for someone other than their child or grandchild, and managing personal care such as giving medications, feeding, dressing, or bathing. We further classified caregivers based on what they reported as the care recipient’s main health problem, long-term illness, or disability: Alzheimer’s disease, dementia, or another cognitive impairment disorder (“caregivers of PWD”) or some other conditions (“caregivers of PWOC”). For this study, we identified 11,466 Baby Boomers who met these criteria as caregivers.

We classified caregivers in terms of their relationship to the care recipient (caring for a parent or parent-in-law; spouse or partner; other family member (grandparent, sibling, or other family member); or non-family member); how long they had been providing care (less than 2 years, 2 years or longer); how many hours, on average, they provided care (less than 20 h/week, 20 h/week or more); and whether they helped with managing household tasks (such as cleaning, managing money, or preparing meals, yes or no).

We used questions from the core BRFSS to classify caregivers’ health and living situations. Specifically, we included self-reported general health status, categorizing it as excellent, very good, or good versus fair or poor. We classified caregivers as experiencing frequent mental distress if they answered 14 or more days [16] to the following question, “Now thinking about your mental health, which includes stress, depression, and problems with emotions, for how many days during the past 30 days was your mental health not good?” We classified respondents as having at least one chronic health condition if they said they had ever been diagnosed with arthritis, cardiovascular disease (angina, stroke, or myocardial infarction), diabetes (excluding gestational or pre-diabetes), cancer (other than skin cancer), or chronic obstructive pulmonary disease, or if they reported they currently had asthma. We required that respondents said yes or no to at least four of the six conditions in order to calculate this variable. We also created variables to indicate whether there were any children under the age of 18 years old living in the household.

### 2.3. Sample of Baby Boomer Caregivers

We included caregivers in the analysis who were classified as Baby Boomers and were not missing responses for the following primary variables of interest: caregivers of PWD status, general health status, frequent mental distress status, and having at least one chronic health condition. We also limited our sample to people who had valid responses for the following covariates included in regression models: sex, education, race/ethnicity, relationship to care recipient, caregiving duration, and caregiving hours/week. Among 11,466 Baby Boomers who were caregivers and responded to the four questions used to classify caregiving status, we excluded 864 people (7.5% unweighted) because of missing information on at least one variable listed above; therefore, our final unweighted sample size of caregivers was 10,602. Respondents who were excluded were more likely to live in lower income households and provide care for a non-relative. Excluded respondents were less likely to be Hispanic and helping with household tasks as part of caregiving. Included and excluded respondents were similar in terms of sex, age group, education, employment, general health status, frequent mental distress status, chronic condition prevalence, caring for a parent, providing care for 20 h/week or more, and caregiving duration.

### 2.4. Statistical Analysis

We calculated weighted prevalence estimates of caregiving among Baby Boomers and weighted prevalence estimates of selected characteristics by dementia status of care recipients among Baby Boomers who were caregivers. Weighting was necessary to assure that the data represent the population from which they were drawn. We used chi-square tests to compare caregivers of PWD and those with PWOC. If the unweighted denominator was <50 or the relative standard error (calculated as weighted standard error divided by weighted percentage multiplied by 100) was >30.0%, we did not report the estimate because these estimates may not be reliable. This decision was informed by a historical practice used for the National Health Interview Survey [17]. We used separate log-binomial regression models to estimate the adjusted prevalence ratios (PR) for having fair or poor health, frequent mental distress, and at least one chronic health condition. We tested each of these models for effect modification by sex by including an interaction between sex and caregiver group in models because we hypothesized that the relationship between being a caregiver and mental and physical health might differ between men and women based on previous research [18]. We adjusted each model for age in years, sex, race category (white, non-Hispanic versus all other groups), educational attainment (high school degree or less versus some college or higher), caregiving duration, caregiving hours/week, and providing household tasks. We considered *p* < 0.05 to indicate statistical significance, including for effect modification. We used survey commands with subpopulation statements as appropriate to restrict to respondents who were Baby Boomers, caregivers, and not missing covariates in Stata version 13.1 (College Station, TX, USA) for all analyses.

## 3. Results

Among the 10,602 Baby Boomers who were caregivers, 15.4% were caregivers of PWD and 84.6% were caregivers of PWOC. Table 1 presents the demographic and health characteristics of Baby Boomers who were caregivers of PWD and caregivers of PWOC. Caregivers of PWD were more likely than caregivers of PWOC to be women (73% versus 64%, *p =* 0.008), and have at least some college education (71% versus 61%; *p* = 0.008). All other sociodemographic and health characteristics of both groups of caregivers were similar.

Parent or parent-in-law was the most commonly reported care recipient relationship for both groups of caregivers, although caregivers of PWD were significantly more likely to be providing care to a parent or parent-in-law than caregivers of PWOC (72% versus 43%, *p* < 0.001, Table 2). In addition, caregivers of PWOC (29%) were significantly more likely to be caring for their spouses or partners compared to caregivers of PWD (11%) (*p* < 0.001). The intensity of care was similar regardless of the care recipient’s diagnosis: most caregivers, on average, provided less than 20 h/week of care (55–56%). However, in terms of the duration of care, caregivers of PWD were significantly more likely to have been providing care for two years or longer (66% versus 55%, *p* = 0.002) than caregivers of PWOC.

Table 3 presents the prevalence ratios (PR) of physical and mental health characteristics comparing Baby Boomers who were caregivers of PWD and caregivers of PWOC. We found no effect modification by sex, so each model is adjusted for sex rather than stratified by sex. The prevalence of self-reported fair or poor health, frequent mental distress, and having at least one chronic condition were not significantly different between caregivers of PWD and those of PWOC (fair or poor health: PR = 0.91, 95% CI: 0.69–1.19, *p* = 0.478; frequent mental distress: PR = 1.04, 95% CI: 0.81–1.34, *p* = 0.738; at least one chronic condition; PR = 1.03, 95% CI: 0.95–1.12, *p* = 0.455). Older age was associated with a higher prevalence of having at least one chronic condition (PR = 1.02, 95% CI: 1.01–1.02, *p* < 0.001) but a lower prevalence of frequent mental distress (PR = 0.97, 95% CI: 0.96–0.99, *p* = 0.001). Sex was significantly associated with one outcome; female caregivers were more likely than male caregivers to experience frequent mental distress (PR = 1.39, 95% CI: 1.13–1.70, *p* = 0.002). Lower educational attainment was associated with fair or poor general health (PR = 1.69, 95% CI: 1.42–2.02, *p* < 0.001) and having at least one chronic condition (PR = 1.11, 95% CI: 1.04–1.18, *p* = 0.002). Non-Hispanic white caregivers were less likely to have fair or poor health (PR = 0.81, 95% CI: 0.67–0.97, *p* = 0.024) but more likely to experience frequent mental distress (PR = 1.51, 95%CI: 1.19–1.91, *p* = 0.004) compared to other racial/ethnic groups of caregivers. Providing care for a parent was consistently associated with a lower likelihood of poor health outcomes (PR range: 0.71–0.88, all *p* ≤ 0.001). However, caregiving for two years or longer was significantly associated with fair or poor general health (PR = 1.27, 95% CI: 1.07–1.51, *p* = 0.007). Providing care for 20 h/week or more was associated with a higher prevalence of fair or poor general health and frequent mental distress (PR = 1.40 and 1.54, respectively, both *p* < 0.001).

## 4. Discussion

Using 2015–2018 BRFSS data (*N* = 10,602), the prevalence of Baby Boomer caregivers of PWD was described, and the physical and mental health of caregivers of PWD was compared to PWOC. We found that Baby Boomers who were caregivers of PWD compared to their counterparts had provided significantly more hours of care for parents/parents-in-law over a longer period of time. This is consistent with previous studies [14] suggesting that Baby Boomers who are caregivers of PWD provide care to an older person whose condition typically does not improve but deteriorates over time. Therefore, their caregiving requires a longer period of time with increasing care needs. On the contrary, caregivers of PWOC tend to provide care for their spouses/partners, who might not be as old as PWD in most cases. Moreover, depending on the health condition of their care recipient, there is a possibility that the conditions of their care recipients could improve.

In our study, there was no difference in physical and mental health between caregivers of PWD and caregivers of PWOC who help to manage personal care. Thus, our hypothesis was not supported. Previous studies have found that caregiving for PWD is psychologically and physically more challenging than caregiving for persons with other types of conditions. Therefore, caregivers of PWD experienced high caregiver burden and depressive symptoms as their care recipient’s behavior problems intensified [19,20] and care recipient’s dementia-specific behavior problems have also negatively impacted the emotional distress of caregivers of PWD [21], as well as lowered caregiver’s quality of life [22].

The majority (72%) of caregivers of PWD in this study were caring for their parents or parents-in-law, which is consistent with many previous studies [19,23]. Across all three outcomes evaluated in this study (i.e., general health, mental distress, and chronic conditions), caring for parents was associated with a lower prevalence of poor health. Previous studies have reported positive effects of caregiving such as a sense of purpose in life [24] and a feeling of usefulness [25]. Filial caregiving by adult children, including care for parents living with dementia [26], may bring a sense of reciprocity between adult children and their parents, greater closeness toward their parent(s), and enhance their sense of responsibility [27,28]. Adult children providing care might find caregiving rewarding despite its potentially intense and demanding nature. In terms of physical health, it is less clear how caregiving, especially caring for a parent, might differentially impact a caregiver’s health [24] because most studies have focused on the mental health of caregivers. However, some previous studies have found negative impact on physical health among caregivers of PWD; older caregivers of PWD had poorer health [29], especially among mentally distressed caregivers of PWD [14]. However, Karg et al.’s study [22] also have reported that, although a significantly higher level of mental distress was found among caregivers of PWD, no significant physical health difference was found between caregivers of PWD and caregivers of all ill patients. The reason could be that because caregiving of PWD requires more intense physical performance, caregivers of PWD may be more physically active and maintain their health in order for them to continue their caregiving [30]. A population-based study has shown that healthier individuals are more likely to take on a caregiving role [31]. Furthermore, other population-based studies have found that non-Hispanic white, female, adult child caregivers who provide care to their parents, in particular, have a significantly lower rate of death compared to non-caregivers [24,32]. Good physical health may be an expectation for being a caregiver so that they can care for others [31]. Adult child caregivers who provide more physically intense, personal care, may need to maintain a physically active lifestyle in order to be able to provide needed hands-on care. Caring for their parent can be a good opportunity for adult children to have closer relationships with their parents, fulfill their filial duty, and benefit their physical and mental health.

Several study limitations should be noted. Because this is a cross-sectional study, causation is unknown. We do not know if caregiving was the cause of any health status or health condition. The chronic health conditions of Baby Boomers who were caregivers of PWD and PWOC were similar, and, indeed, it is possible that people who take on the caregiving role do so, in part, because they are in good health. The BRFSS Caregiver Module interviews caregivers only, providing detailed information on the caregiving situation and the caregivers’ health and well-being. More detailed information about the care recipient such as age, cognition, health status, and behaviors was not available because they were not interviewed as part of the BRFSS protocol. Caregivers could report only a single health condition for the perceived main reason the care recipient needs care. It is possible that some care recipients classified as PWOC may have multiple reasons for needing care that also have dementia or cognitive decline. Despite these limitations, this study included a very large, representative sample of community-dwelling adults from 44 states, the District of Columbia, and Puerto Rico.

## 5. Conclusions

Baby Boomers who are caregivers of PWD reported similar physical and mental health compared to caregivers of PWOC. The majority of caregivers do have chronic health conditions such as arthritis, asthma, cardiovascular disease, diabetes, or COPD, and experienced mental distress. Additional longitudinal data are needed to more fully understand the causal effects of providing care including long-term health impacts, especially in older caregivers or those with multiple chronic health conditions. As Baby Boomers continue to grow older, efforts are necessary to support their continued caregiving role, protect their health status, and promote self-care as many Baby Boomers are older adults themselves. They, like caregivers of all ages, need to take care of their own health and psychological well-being, too. The importance of maintaining the health and well-being of Baby Boomers who are caregivers of PWD and PWOC should not be underestimated as well as the significant role they play in supporting the health, well-being, and independence of their care recipient.

## Figures and Tables

**Table 1:** Demographic and Health Characteristics of Baby Boomer Caregivers of Persons with Dementia (PWD) and Caregivers of Persons with Other Conditions (PWOC), Behavioral Risk Factor Surveillance System, 2015–2018.

Variable	Category	Caregivers of Persons with Dementia (PWD) (*n* = 1661) Weighted %	Caregivers of Persons with Other Conditions (PWOC) (*n* = 8941) Weighted %	*p*-Value ^1^
Sex	Female	72.5	64.2	0.008
Age	50–54	20.6	24.8	0.358
	55–59	25.9	26.3	
	60–64	27.4	27.9	
	65–72	26.1	21.0	
Mean Age	Mean (SD)	60.1 (6.4)	59.5 (6.5)	0.155
Race/ethnicity	Black only, non-Hispanic	15.8	11.2	0.254
	White only, non-Hispanic	72.1	71.4	0.872
	Any race, Hispanic	8.8	11.7	0.209
	Asian or Pacific Islander, non-Hispanic	1.1	1.7	0.426
	Other race or multiracial, non-Hispanic	2.3	4.0	0.093
Education	Less than high school	4.8	10.2	0.008
	High school degree or equivalent	23.9	29.0	
	Some college	41.9	36.7	
	College graduate	29.4	24.1	
Employment Status	Employed/Self-employed	49.7	49.8	0.956
	Out of work	5.4	5.2	0.852
	Homemaker	5.8	6.8	0.452
	Retired	28.7	24.2	0.265
	Unable to Work	10.1	13.3	0.214
	Missing	0.3	0.6	--
Annual Household Income	Less than $15,000	6.9	10.0	0.218
	$15,000–24,999	10.8	15.6	
	$25,000–49,999	21.0	22.6	
	$50,000–74,999	19.4	16.1	
	$75,000 or more	30.3	25.7	
	Missing	11.7	10.1	--
General Health Status	Excellent, very good or good	78.7	73.1	0.100
	Fair or poor	21.3	26.9	
Frequent Mental Distress	≥14 days of poor mental health in the past 30 days	14.7	15.5	0.660
Chronic Health Conditions	Arthritis	49.7	46.0	0.340
	Asthma (current only)	15.2	11.0	0.148
	Cancer (except skin)	9.5	10.7	0.567
	Cardiovascular disease ^2^	14.4	13.3	0.719
	Diabetes (except gestational)	18.6	19.3	0.810
	Chronic obstructive pulmonary disease	11.7	12.7	0.751
Any Chronic Condition	At least one of the chronic condition	65.6	66.3	0.834

^1^*p*-Value comparing caregivers of people with dementia to other caregivers based on the chi-square test of weighted proportions or *t*-test of difference in means (age only). ^2^ Cardiovascular disease includes angina, stroke, or myocardial infarction—Not reported because relative standard error >30.0, indicating unstable estimate.

**Table 2:** Characteristics of Baby Boomer Caregivers of Persons with Dementia (PWD) and Caregivers of Persons with Other Conditions (PWOC), Behavioral Risk Factor Surveillance System, 2015–2018.

Variable	Category	Caregivers of Persons with Dementia (PWD) (*n* = 1661) %	Caregivers of Persons with Other Conditions (PWOC) (*n* = 8941) %	*p*-Value ^1^
Relationship of care recipient	Parent or parent-in-law	72.2	42.7	<0.001
	Spouse or partner	10.7	29.3	<0.001
	Other relative	13.5	15.7	0.635
	Non-relative	3.6	12.3	<0.001
Caregiving Duration	<2 years	34.2	45.1	0.002
	≥2 years	65.8	54.9	
Caregiving Hours	<20 h/week	54.6	56.1	0.682
	≥20 h/week	45.4	43.9	
Helps with Household Tasks	Yes	91.8	90.8	0.494

^1^*p*-Value comparing caregivers of people with dementia and other caregivers based on the chi-square test of weighted proportions.

**Table 3:** Prevalence ratios of fair or poor health, frequent mental distress, or having at least one chronic condition for caregivers of persons with dementia (PWD) based on multivariable log-binomial regression models, Behavioral Risk Factor Surveillance System, 2015–2018.

Variable	Category	Fair or Poor General Health PR (95% CI) *p*-Value	Frequent Mental Distress PR (95% CI) *p*-Value	At least 1 Chronic Condition ^1^ PR (95% CI) *p*-Value
Caregiver Type	Caregivers of Persons with Dementia (PWD)	0.91 (0.69–1.19) 0.478	1.04 (0.81–1.34) 0.738	1.03 (0.95–1.12) 0.455
	Caregivers of Persons with Other Conditions (PWOC)	1.0 (Reference)	1.0 (Reference)	1.0 (Reference)
Age	Per year	0.99 (0.98–1.01) 0.399	**0.97** **(0.96–0.99)** **0.001**	**1.02** **(1.01–1.02)** **<0.001**
Sex	Female	0.92 (0.78–1.08) 0.301	**1.39** **(1.13–1.70)** **0.002**	1.00 (0.94–1.07) 0.914
	Male	1.0 (Reference)	1.0 (Reference)	1.0 (Reference)
Highest education level	High school degree or less	**1.69** **(1.42–2.02)** **<0.001**	**1.14** **(0.92–1.41)** **0.225**	**1.11** **(1.04–1.18)** **0.002**
	Some college or more	1.0 (Reference)	1.0 (Reference)	1.0 (Reference)
Race/ethnicity category	White, non-Hispanic	**0.81** **(0.67–0.97)** **0.024**	**1.51** **(1.19–1.91)** **0.001**	1.01 (0.92–1.11) 0.811
	All other groups	1.0 (Reference)	1.0 (Reference)	1.0 (Reference)
Care recipient relationship to caregiver	Parent	**0.72** **(0.59–0.87)** **0.001**	**0.71** **(0.58–0.87)** **0.001**	**0.88** **(0.82–0.95)** **<0.001**
	Non-parent (other relative or friend)	1.0 (Reference)	1.0 (Reference)	1.0 (Reference)
Duration of care	<2 years	1.0 (Reference)	1.0 (Reference)	1.0 (Reference)
	≥2 years	**1.27** **(1.07–1.51)** **0.007**	1.05 (0.86–1.29) 0.632	1.04 (0.98–1.12) 0.103
Average hours of care per week	<20 h	1.0 (Reference)	1.0 (Reference)	1.0 (Reference)
	≥20 h	**1.40** **(1.18–1.67)** **<0.001**	**1.54** **(1.25–1.89)** **<0.001**	1.04 (0.98–1.11) 0.206

*N* = 10,602 for all three models; Bold text indicates statistically significant estimates (*p* < 0.05). ^1^ Chronic conditions include arthritis, current asthma, non-skin cancer, cardiovascular disease, diabetes, and chronic obstructive pulmonary disease.

## Data Availability

The Behavioral Risk Factor Surveillance System (BRFSS) is a publicly available dataset. The information is cited in the manuscript.
